# Dr. William B. Southard

**Published:** 1904-04

**Authors:** 


Dr. William B. Southard, of Kalamazoo, Mich., a graduate of the University of Buffalo, 1859, died February 21, 1904, aged 81 years.

